# A review of dominant lactic acid bacteria strains and inoculation fermentation for fermented mustard plant

**DOI:** 10.3389/fmicb.2026.1799812

**Published:** 2026-03-20

**Authors:** Yongtong Zhou, Angye Cai, Zhen Mo, Nan Diao, Jingyi Huang, Baijun Long, Siwei Shang, Wenzhong Hu, Ke Feng, Sarengaowa

**Affiliations:** 1 College of Life Science, Zhuhai College of Science and Technology, Zhuhai, China; 2 Faculty of Medicine, Macau University of Science and Technology, Macau, China

**Keywords:** dominant strains, fermentation mechanism, isolation and screening, LAB identification, mustard plant

## Abstract

As a member of the *Brassicaceae* family, mustard plant is an annual herbaceous plant rich in diverse nutrients. Traditionally, it is often processed through spontaneous fermentation to improve its unique spicy flavor. However, spontaneous fermentation has certain problems, such as a long cycle, unstable quality, and safety risks. To promote the standardization and efficiency of mustard plant fermentation, the screening and application of inoculation fermentation using dominant strains have become research hotspots. This study reviews the isolation, screening, and identification methods of dominant strains in mustard plant fermentation (such as *Lactiplantibacillus plantarum*, *Weissella*, *Lactobacillus brevis*, and yeast), including traditional culturing technology, physicochemical analysis, and molecular biology technology (such as Illumina Miseq high-throughput sequencing and 16S rDNA sequencing). Research shows that lactic acid bacteria have outstanding acid production, acid and salt resistance, nitrite degradation, and antioxidant and flavor regulation abilities during the fermentation process, which significantly improve the safety, flavor, and quality of the products. The mechanism of action of dominant strains in the fermentation process is further discussed, including organic acid metabolism, amino acid transformation, volatile flavor formation, and microbial community dynamic evolution. This article provides a theoretical basis and technical reference for the change from traditional natural fermentation to high-quality and high-efficiency inoculation fermentation of mustard plant.

## 1 Introduction

Mustard plant is an annual herbaceous plant from the family Cruciferae, which is rich in vitamins, minerals, dietary fiber, and other nutrients. However, due to its unique spicy flavor, people usually choose to ferment and process mustard plant before eating (Yu et al., 2022a,b). The traditional fermentation process of mustard plant mainly relies on the spontaneous fermentation of microorganisms, mainly the process of breaking organic macromolecules into simple molecules. Under saline conditions, the microorganisms attached to the surface of mustard plant (such as lactic acid bacteria (LAB) and yeasts) carry out a series of lactic acid fermentation and esterification reactions to produce organic acids, free amino acids (FAAs), and volatile substances (Liu et al., 2025), thus giving the product a unique flavor and extending the shelf life (Peng et al., 2025).

However, the current widely used traditional natural fermentation has many limitations, including the long fermentation cycle, unstable quality, and potential safety risks (Ruiz-Barba et al., 2023). During natural fermentation, mustard plants are susceptible to contamination with miscellaneous bacteria, leading to complex and diverse microorganisms being carried by mustard plant products, which is not conducive to quality control (Hang et al., 2022). To realize standardized production and improve production efficiency and product quality, more and more people choose inoculation and fermentation. Therefore, using the dominant strains as starters to direct the fermentation process is a key strategy to optimize mustard plant fermentation. Several studies have found that high-throughput sequencing (HTS), high-performance liquid chromatography (HPLC), and gas chromatography–mass spectrometry (GC–MS) methods can identify dominant strains in natural fermented mustard plants, which have considerable potential in improving product quality (Wang D. D. et al., 2022; Yu et al., 2021; Zhang et al., 2021). The selected dominant strains can quickly lead the fermentation process, resulting in the ideal pH value and flavor formation being reached, and thus improving the content of flavor metabolites. Regarding safety, this can effectively reduce the nitrite content, prolonging the shelf life of products.

This study aims to summarize the isolation, screening, and identification methods of dominant strains; analyze various potential candidate microorganisms with fermentation characteristics; and further explore fermentation mechanisms to better understand the dominant strains in inoculation fermentation processes and provide a reference for the transition from traditional spontaneous fermentation to high-quality and efficient inoculation fermentation.

## 2 Isolation, screening, and identification of dominant strains

**Figure 1:**
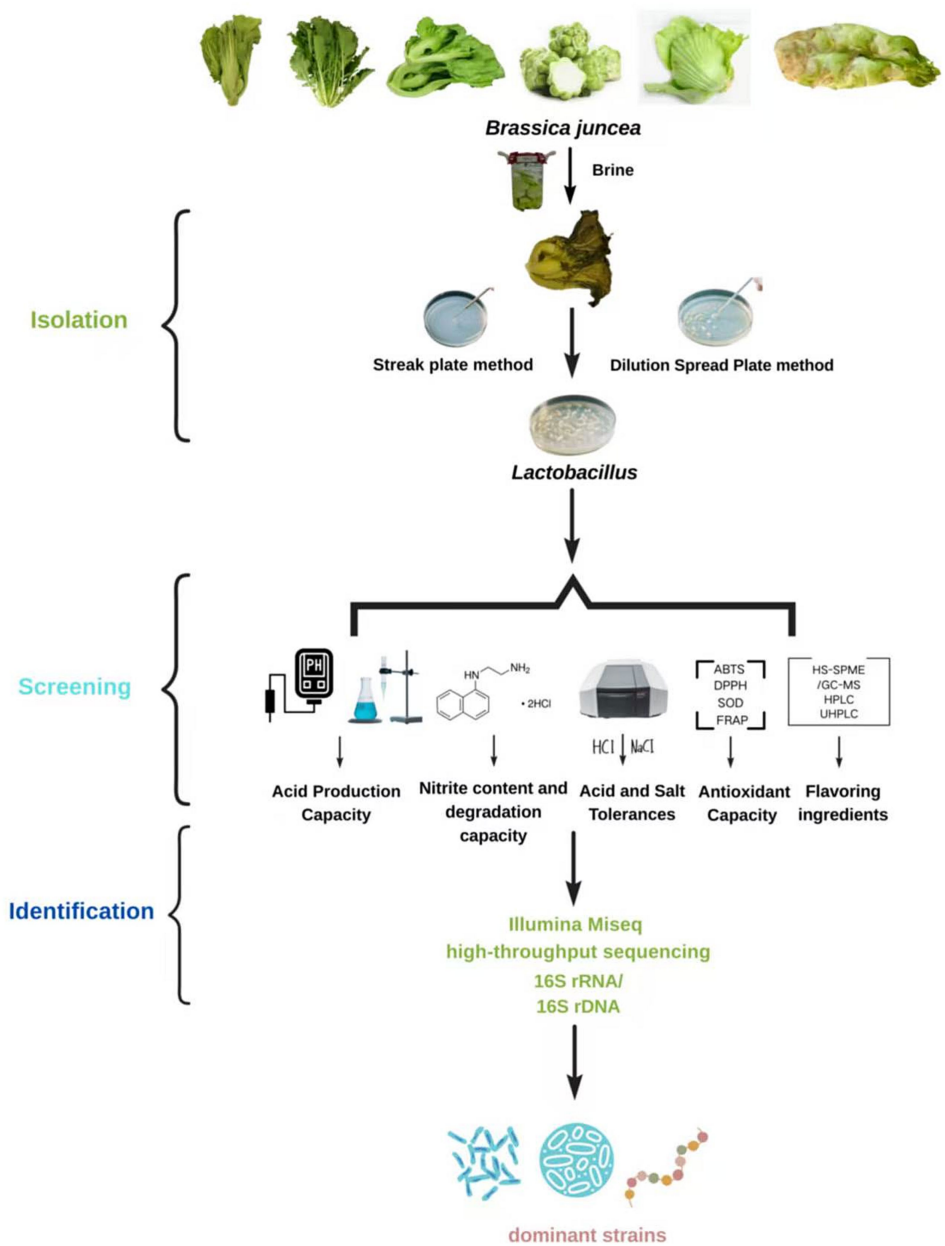
Flow chart of lactic acid bacteria isolation, screening, and identification. HS-SPME/GC–MS refers to headspace solid-phase microextraction/gas chromatography–mass spectrometry; HPLC refers to high-performance liquid chromatography; UHPLC refers to ultra high-performance liquid chromatography.

The acquisition of superior bacterial strains typically involves three key steps: isolation, screening, and identification. Isolation involves obtaining pure cultures from fermented mustard plant or its juice using methods like streak plate method or dilution spread plate method. Screening serves as the core process, where strains with superior performance are selected based on their physicochemical characteristics. Finally, identification confirms the strains’ taxonomic status through colony morphology, microscopic examination, and molecular biology techniques. This workflow aims to efficiently isolate pure strains with application potential from complex microbial populations (Figure 1).

### 2.1 Isolation

The isolation of potential fermentation strains from fermented mustard plant serves as the first critical step in screening for superior bacterial strains. Commonly used methods include the streaking method and the dilution plate technique (Table 1). Liu et al. (2025) employed the streak plate method, sampling both commercial and homemade mustard plant. They added pickling juice to specific culture media at predetermined ratios, followed by preliminary cultivation before selecting individual colonies for further streaking to achieve strain isolation and purification. Kong et al. (2025) and Wu et al. (2021) also used the streak plate method, performing dilutions in juice samples obtained from mustard plant and inoculating the diluted solutions onto selective media like MRS agar. The cultures were incubated under anaerobic conditions at 37 °C for 48 h. Notably, the latter’s MRS agar contained 1% CaCO_3_. After streaking more than three times on MRS agar, Gram-positive white colonies with calcium-soluble rings were selected for isolation through morphological observation and Gram staining. The size of these calcium-soluble rings could also indicate acid-producing capacity. This method combined biochemical techniques such as Gram staining and hydrogen peroxide tests to further isolate potential fermentation strains during the separation process, a crucial step absent in the study by Kong et al. (2025). Xiong Y. B. et al. (2024) employed the dilution spread plate method in their experiment, where they prepared gradient dilutions of mustard plant fermented broth and cultured the mixture on MRS solid medium supplemented with 2% CaCO_3_. This approach shares similarities with that of Wu et al. (2021), as both experiments involved isolating pure cultures from milky white single colonies exhibiting distinct transparent calcium-soluble zones, followed by Gram staining and catalase assays. However, Wu’s methodology differed from Xiong’s by using the streak plate method instead of the plate spreading method. Existing research has predominantly utilized the streaking method for strain isolation, while experiments employing the dilution spread plate method remain relatively uncommon.

**Table 1:** Methods for bacterial isolation and screening.

*Brassica juncea* region	Dissociation method	Screening indicators	Screening method	Dissociation strain	References
Sichuan, Guizhou, Yunnan, and Guangdong	Streak plate method	Acid production capacity	BCG acid–base indicator titration method	*Lactiplantibacillus plantarum* CS8, *Saccharomyces cerevisiae* CX1, *Pichia manshurica* CX3, *Zygosaccharomyces bisporus* CX2, *Kazachstania bulderi* CX4	Liu et al. (2025), Chen et al. (2006)
		Nitrite content, degradation capacity	Naphthyl enediamine hydrochloride colorimetric method		
		Acid resistance, salt tolerance	pH measurement, strain salt tolerance experiment		
		Flavor components	HS-SPME/GC–MS, HPLC		
Ningbo, Zhejiang Province	Streak plate method	Acid production capacity	FE28 pH meter	*Weissella cibaria* LAB1, LAB3, *Leuconostoc mesenteroides* LAB2, LAB4	Kong et al. (2025), Hou et al. (2023)
		Nitrite degradation capability	Microbial degradation method		
		Acid resistance and salt tolerance	pH measurement and strain salt tolerance experiment		
		Antioxidant capacity	DPPH free radical scavenging		
		Antibacterial capability	Agar well diffusion assay		
Guiyang, Anshun, Zunyi	/	Acid production capacity	pHS-3C pH meter	*Lactobacillus curvatu*, *Lactiplantibacillus plantarum*, *Lactobacillus hokkaidonensis*, *Lactobacillus parabuchneri*, *Lactobacillus buchneri*, *Lactobacillus brevis*, *Lactobacillus sakei*, *Lactobacillus fermentum*, *Lactobacillus coryniformis*, *Lactobacillus delbrueckii*.	Li L. L. et al. (2024)
		Total acid content	Acid–base titration		
		Nitrite content	Naphthyl enediamine hydrochloride colorimetric method		
		Flavor components	HS-SPME		
Yueyang, Hunan Province	Dilution spread plate method	Acid production capacity	FE28 pH meter	*Lactobacillus* sp. KKSX-1~KKSX-12, KKSD-1~KKSD-7, SYS-1~SYS-9	Xiong Y. B. et al. (2024)
		Nitrite degradation capability	Microbial degradation method		
		Acid resistance and salt tolerance	Acid-resistant survival counting method, bile salt-resistant survival rate counting method		
		Antibacterial capability	Oxford cup diffusion method		
Sichuan, Guangdong, Guizhou, Fujian, Jiangsu, Jiangxi Provinces	Streak plate method	Nitrite content	Spectrophotometry	*Lactobacillus plantarum* MLG5-1 GZ-2, *Lactobacillus* sp. KLDS 1.0702 (JX-3), *Lactobacillus brevis* 6,323 (SC-2), *Leuconostoc carnosum* JB16 (GD-2)	Yu et al. (2021)
		Histamine content	HPLC		
Huarong, Xinning, Baojing, Hunan Province	Streak plate method	Acid production capacity	pHS-3C pH meter	*Lactiplantibacillus plantarum* LJ036, LJ065, QT038, JC091, JC045	Lai et al. (2026)
		Nitrite content, degradation capacity	Microbial degradation method		
		Acid resistance and salt tolerance	pH measurement and strain salt tolerance experiment		
		Antioxidant capacity	DPPH free radical scavenging		
		Antibacterial capability	Agar diffusion method		
		Flavor components	SPME-GC–MS		

### 2.2 Screening

Vegetable fermentation strains must meet three criteria: superior fermentation performance, an excellent safety profile, and notable probiotic characteristics (Liu et al., 2025). Physicochemical analysis serves as a critical evaluation method for assessing both the fermentation process and microbial performance. These parameters not only reflect the chemical transformations occurring during fermented mustard plants but also directly determine the final product’s flavor profile, safety standards, and nutritional value. Therefore, when screening dominant strains, comprehensive evaluations should be conducted through testing key physicochemical indicators, including acid production capacity, nitrite content and degradation efficiency, acid/salt tolerance, antioxidant properties, and flavor component profiles to holistically assess different bacterial strains’ fermentation capabilities (Table 1).

#### 2.2.1 Acid production capacity

In fermentation processes, acid production capacity serves as a critical indicator for evaluating fermentation efficiency and product quality. To accurately measure this capability in fermented mustard plant, various methods can be employed, including direct pH measurement, acid–base indicator titration, and potentiometric titration. Experiments by Kong et al. (2025) and Yu et al. (2022b) both utilized the direct pH-metering method to measure the pH value of the fermentation broth, which is straightforward and convenient, making it the most commonly used approach for pH value detection. Studies have shown that the pH value of fermented vegetables generally decreases during the fermentation process, which is consistent with the results of Ngamsamer et al. (2024). The acid–base indicator titration method is used to assess titratable acidity (TA) by titrating the filtrate with sodium hydroxide solution, using lactic acid as the standard solution for calculation (Chien et al., 2023). However, since this method cannot distinguish between volatile and non-volatile acids, the comprehensive detection of total acid content requires more advanced techniques, such as potentiometric titration or spectrometry, to accurately determine the concentrations of all acidic components (Jiang et al., 2023).

Some scholars have pointed out that traditional methods for screening, isolating, and purifying bacterial strains from fermented foods are generally inefficient, time-consuming, and labor-intensive (Garmasheva et al., 2019). Therefore, there is a need for a rapid and effective screening method. Liu et al. (2025) performed an experiment utilizing bromocresol green (BCG), which is pH-sensitive and suitable for evaluating acidification capacity within specific pH ranges, as the screening indicator. By combining BCG, microplate readers, and high-throughput screening equipment, they developed a rapid and effective preliminary screening method for acid-producing strains. In this method, color changes are observed in two culture media under different pH values, and corresponding absorbance values at color transitions are detected to characterize acid production capabilities through distinct absorbance levels. Although this approach significantly improves screening efficiency, the small culture volume in microtiter plate systems makes it susceptible to interference. Consequently, this method serves as an initial screening tool for acid-producing microorganisms, requiring further re-screening to identify strains with exceptional acid production perf.

#### 2.2.2 Nitrite content and degradation capacity

Nitrite content serves as a critical food safety indicator during fermentation processes. Research indicates that nitrites in fermented mustard plant may pose potential health risks (Kim et al., 2017). To ensure the safety of fermented mustard plant and human health, it is essential to monitor nitrite levels during fermentation and implement effective measures to reduce them. The determination of nitrite content typically employs the naphthyl enediamine hydrochloride colorimetric method (Chen et al., 2006). This method utilizes the reaction between p-aminobenzenesulfonic acid and naphthyl enediamine hydrochloride, where the resulting product exhibits absorbance at a wavelength of 538 nm. Standard curves are plotted using these measurements to calculate nitrite concentrations in fermented samples. Liu et al. (2025) found that the nitrite content in naturally fermented samples was significantly higher than in optimized fermentation, co-fermentation, and two-phase fermentation. During fermentation, nitrite levels generally showed an initial rise followed by a decline, which may be attributed to the low LAB content in the early stage of fermentation, potentially accelerating nitrite accumulation. However, as LAB acidified the fermentation environment, they inhibited the growth of nitrate-reducing bacteria, ultimately leading to a decrease in nitrite content (Chen et al., 2018). Notably, spontaneous fermentation exhibited a rebound after the nitrite content declined, while optimized fermentation, co-fermentation, and two-phase fermentation showed a gradual stabilization post-decline. This may be because optimized fermentation processes and the inoculation of specific strains effectively reduced nitrite levels during fermentation. The experiments by Jiang et al. (2023) yielded similar results to Liu’s study, revealing that nitrite levels continued to decline for the first 20 days before stabilizing. Compared with naturally fermented samples, inoculated fermentation resulted in significantly lower nitrite content. Additionally, most studies indicate that after fermentation, the nitrite content in pickled vegetables generally does not exceed the maximum allowable nitrite level in preserved vegetables, which should not exceed the 20 mg/kg requirement stipulated by China’s national standards (Jiang et al., 2023; Liu et al., 2024; NHC and SAMR, 2022).

Studies have shown that inoculation with LAB can effectively reduce nitrite levels (Lee K. W. et al., 2016). To evaluate the nitrite-degrading capacity of LAB during fermentation, Hou et al. (2023) conducted an experiment where seed liquid from *Allium chinense* bulbs was inoculated into a nitrite-containing medium. After cultivation and centrifugation, the supernatant was mixed with a nitrite indicator to assess its degradation capability through color changes in the medium. Similarly, this method can serve as a reliable approach for evaluating the nitrite-degrading performance of LAB in fermented mustard plant.

#### 2.2.3 Acid and salt tolerances

Acid and salt tolerance are important factors affecting the quality and preservation of fermented mustard plant. Acid tolerance mainly refers to the ability of LAB to adapt to acidic environments during fermentation, while salt tolerance involves the growth of fermenting microorganisms under high salt concentrations.

In some experiments, the absorbance of bacterial suspension was measured using UV spectrophotometry to assess the acid and salt tolerance of LAB. Acid tolerance serves as a crucial indicator for evaluating the quality of fermented mustard plant, as LAB activity directly impacts the formation of the sour flavor. Researchers suggest that high-quality fermentation agents should withstand pH levels around 4.0 to enhance fermentation efficiency and shorten processing time. Kong et al. (2025) demonstrated that *Weissella* LAB1 and LAB3, and *Leuconostoc mesenteroides* LAB2 and LAB4 showed inhibited growth at pH 2.0 and 3.0. Except for LAB4, the other three strains exhibited robust growth at pH 4.0. Similarly, Lai et al. (2026) revealed that five LAB grew well at pH 4.0 but experienced growth inhibition below pH 3.5. The optimal salt concentration for sauerkraut is approximately 0.7 (3.0%), whereas kimchi requires 3.0 (5.0%) (Lee et al., 2024). Salt tolerance, as a critical indicator for evaluating microbial growth capacity in high-salt environments, not only influences the fermentation quality of mustard raw materials but also directly affects the activity of lactic acid bacteria. Studies have demonstrated that different lactic acid bacteria exhibit varying degrees of salt tolerance. Hu et al. (2022) found that LAB grew well at NaCl concentrations of 0–4%, but could hardly grow when exceeding 6%. Kong et al. (2025) demonstrated that bacterial suspension absorbance values exhibited a declining trend with increasing salt concentration, and strain growth was significantly inhibited when salt concentration exceeded 8%. Similarly, Lai et al. (2026) confirmed that LAB displayed strong salt tolerance when salt concentration was below 8%. In summary, lactic acid bacteria exhibit optimal growth and strong acid resistance at a pH of approximately 4.0, with a salt tolerance threshold typically below 8%. Beyond this range, their growth activity is significantly inhibited. These findings, respectively, demonstrate the inhibitory effects of acidic environmental stress and osmotic pressure stress on cell growth. However, practical mustard fermentation applications often present more complex scenarios. Industrial-grade salt is commonly used in production, with concentrations far exceeding those of laboratory-purified NaCl. Studies indicate that high salt concentrations can lead to bacterial dehydration, metabolic disorders, and even cell death. *Lactobacillus* can resist salt stress by regulating compatibility solutes, glycolytic enzymes, heat shock proteins, and ion balance (Li Z. X. et al., 2023). Li M. et al. (2019) found that *Lactobacillus plantarum* FS5-5 can cope with high-salt stress by upregulating multiple metabolic pathways, including the coordinated regulation of amino acid metabolism, carbohydrate metabolism, and the expression of cell wall synthesis-related proteins (peptidoglycan hydrolases), thereby maintaining cellular homeostasis and growth.

In addition to measuring the absorbance of fermented bacterial cultures, simulating the human gastrointestinal environment to evaluate LAB’s acid and salt tolerance has become a standard laboratory method. Wu et al. (2022) demonstrated that *Lactiplantibacillus plantarum* may enhance EPS production by upregulating EPS synthesis genes under moderate acid stress (e.g., pH 3.0), thereby improving biofilm formation and altering cell surface charge to resist acid stress. This reveals that EPS synthesis is one of the acid tolerance mechanisms in LAB. Wang S. Y. et al. (2024) demonstrated that *Lactiplantibacillus plantarum* Z22 exhibits certain resistance at pH 2.5. It is crucial that ingested LAB survive through the human digestive tract to function properly (Kim H. et al., 2022). Studies show that *Lactiplantibacillus plantarum* maintains high acid resistance even after 24 h in artificial gastric fluid (Wu et al., 2021; Son et al., 2017). Notably, *Lactiplantibacillus plantarum* Z22 demonstrates enhanced survival under 0.3% bile salt conditions, a finding corroborated by Lee K. H. et al. (2016) experiments. Studies have demonstrated that LAB collaboratively respond to bile salt stress through multidimensional mechanisms. Firstly, the cell wall serves as the first line of defense against bile salts. LAB resist attacks by thickening the cell wall and secreting extracellular polysaccharides (EPS) and S-layer proteins. Secondly, LAB actively pump bile salts, which have passively diffused into the cytoplasm, out of the cell via membrane-associated efflux pumps, thereby reducing intracellular bile salt concentration. Thirdly, LAB produce bile salt hydrolase (BSH) to catalyze the decoupling reaction of conjugated bile salts, decreasing their toxicity and solubility, thus mitigating damage to the cell membrane. Additionally, since bile salts induce oxidative stress and generate reactive oxygen species (ROS), leading to DNA or protein damage, LAB upregulate the expression of chaperone proteins to repair damaged DNA and proteins, maintaining essential cellular life activities. These mechanisms exhibit distinct strain-specificity (Pan et al., 2021). Wu et al. (2021) evaluated bacterial viability in acidic and neutral environments using colony counts in simulated gastric fluid. The results revealed that most strains exhibited strong acid and salt tolerance.

With the widespread application of fermentation agents in the food industry, the acid and salt tolerance of relevant strains has become a key research focus (Chalas et al., 2016). Therefore, acid and salt resistance are crucial characteristics of probiotics. Through comprehensive evaluation methods, we can more accurately analyze the acid and salt tolerance of LABs during the fermentation of mustard plant, which helps in screening out high-quality LAB strains that can effectively promote fermentation.

#### 2.2.4 Antioxidant capacity

During the fermentation of mustard plant, the antioxidant capacity of LAB not only affects their own survival rate but also directly impacts the quality and nutritional value of fermented vegetables. Since free radicals can bind to cells and cause damage, leading to various diseases (Sunthornthummas et al., 2025), scavenging free radicals serves as a crucial indicator and primary method for evaluating antioxidant capacity in fermented vegetables. Most experiments assess antioxidant activity through multiple metrics, including 2,2-diphenyl-1-picrylhydrazyl (DPPH) free radical scavenging activity, 2,2′-Azino-bis(3-ethylbenzothiazoline-6-sulfonic acid) (ABTS) free radical scavenging activity, superoxide dismutase (SOD) activity, fructose reducing antioxidant power (FRAP) determination, and hydroxyl radical scavenging capability. Given that a single method cannot comprehensively evaluate LAB’s antioxidant capacity, researchers typically employ 2–3 combined methods for integrated assessment.

The antioxidant capacity of LAB varies significantly between different strains. Certain LAB exhibit strong antioxidant effects that help to reduce stress-induced oxidative damage in the body (Noureen et al., 2019). Some studies have investigated antioxidant activity by inoculating a single LAB strain. In their research (Park et al., 2023), Sang-Kyu Park et al. employed three methods-DPPH free radical scavenging activity, ABTS free radical scavenging activity, and SOD activity-to comprehensively evaluate the antioxidant properties of *Pediococcus pentosaceus* isolated from Jangajji, a traditional Korean fermented kimchi. When inoculated into broccoli for fermentation, they observed that DPPH scavenging activity and SOD levels initially increased but then declined with extended fermentation time, while ABTS values showed a gradual upward trend. Lee and Kim (2019) similarly used DPPH and SOD assays but additionally applied FRAP measurement to assess the antioxidant activity of *Leuconostoc mesenteroides* MKSR isolated from Korean kimchi. Their study revealed that MKSR demonstrates robust antioxidant capabilities, showing higher DPPH scavenging activity and FRAP levels in both whole cells and cell-free suspensions compared with other LAB. SOD, as an antioxidant enzyme, is considered the most crucial defense system against oxidative stress in LABs, playing a primary role in maintaining balanced redox states (Bajpai et al., 2016; Wu et al., 2012). Some experiments have compared antioxidant capabilities by inoculating different types of LAB. According to the experiment by Kong et al. (2025), a comparative analysis of *Weissella cibaria* (LAB1, LAB3) and *Leuconostoc mesenteroides* (LAB2, LAB4) revealed that the *Weissella cibaria* strain LAB1 exhibited the highest DPPH radical-scavenging rate, demonstrating the most potent antioxidant capacity among all the tested strains. Similarly, Wu et al. (2021) experiment revealed that of the three LAB strains AWP4, AWP7, and AWP11, AWP4 exhibited strong antioxidant activity. This strain was identified as *Lactiplantibacillus plantarum*, with both bacterial genera being dominant strains in the samples.

Various conditions may also influence the free radical scavenging capacity. (Li H. et al., 2023) conducted a comparative study between three groups of *Lactobacillus sakei* MS103: cultured, heat-inactivated, and live cultures. The results showed that the live *Lactobacillus sakei* MS103 group exhibited higher DPPH free radical scavenging activity, though with minimal impact on ABTS. All three groups demonstrated excellent free radical scavenging capabilities. Regarding hydroxyl radical scavenging, the cultured *Lactobacillus sakei* MS103 group outperformed both the live and heat-inactivated groups. Zhou H. C. et al. (2023) compared antioxidant activities before and after pickling, finding no statistically significant difference in free radical scavenging capacity between pre-fermentation and post-fermentation stages. However, FRAP levels were significantly higher in pre-fermented pickles than in post-fermented ones. Researchers speculate that factors such as fermentation temperature, environmental conditions, and phenolic compounds in fresh vegetables may influence antioxidant activity during the process. Furthermore, Zhou H. C. et al. (2023) compared the antioxidant effects of different solvent extracts (ethanol, water, and hot water) on fermented pickles. The results showed that ethanolic extract demonstrated significantly higher DPPH free radical scavenging activity, ABTS free radical scavenging activity, and FRAP levels compared with water extracts (*p* < 0.05). Specifically, hot water extracts exhibited superior ABTS and FRAP activities over water extracts (*p* < 0.05). Among the three types of pickled vegetable extracts, alcohol extracts exhibited the highest antioxidant capacity. Zhou et al. attributed this to the high content of total phenols, flavonoids, and sugars in alcohol extracts, a finding consistent with both Park et al. (2011) and Nguyen and Nguyen (2024). Park et al. (2011) reported that solvent-based extracts (methanol, ethanol, and water) containing phenolic compounds, vitamin C, and phenolic acids were closely associated with DPPH-free radical scavenging. Nguyen and Nguyen (2024) further suggested strong correlations between phenolic compounds and FRAP values, as these substances can eliminate free radicals or inhibit metal ions (Ahmed et al., 2020; Hsieh et al., 2021), thereby enhancing antioxidant activity.

It is widely recognized that highly reducing materials can act as antioxidants. Therefore, indicators such as DPPH, ABTS, SOD, FRAP, and hydroxyl radicals can be used to evaluate the antioxidant capacity of fermented or unfermented vegetables (Zhu et al., 2020). However, factors influencing antioxidant activity extend beyond LAB inoculation efficiency, single or mixed bacterial strains, fermentation temperature, and environmental conditions. We speculate that during the entire fermentation process, the growth and reproduction of LAB generate metabolic byproducts, including various enzymes, phenolic compounds, and total flavonoids, which may collectively enhance the antioxidant capacity of the fermented broth.

#### 2.2.5 Flavoring compounds

Both volatile and non-volatile compounds contribute to the distinctive flavor profile of fermented mustard plant. Volatile components primarily include alcohols, aldehydes, ketones, esters, sulfur-containing compounds, acids, and heterocyclic compounds (Xiao et al., 2018), while non-volatile substances mainly consist of organic acids, free amino acids, sugars, and biogenic amines. These elements work synergistically to create the unique aroma and texture of the fermented mustard plant. To accurately detect flavor components in fermented mustard plant, analytical methods such as gas chromatography–mass spectrometry (GC–MS) and high-performance liquid chromatography (HPLC) are commonly employed (Wu et al., 2015; Xiong et al., 2016).

Headspace solid-phase microextraction/gas chromatography–mass spectrometry (HS-SPME/GC–MS) enables the efficient separation, identification, and extraction of volatile components. Li L. L. et al. (2024) detected 190 volatile compounds in 12 samples of fermented mustard plant from Guizhou Province, while Zhang et al. (2021) identified 62 volatile compounds in samples of fermented mustard plant from Hangzhou City, Zhejiang Province. Analysis revealed that isothiocyanates were predominantly recognized as the primary odor-active substances in various varieties of fermented mustard plant. Their abundant content is considered crucial for preserving the distinctive flavor profile of pickled vegetables (Liu et al., 2025).

Organic acids are a crucial non-volatile flavor component in fermented foods (Shang et al., 2022). HPLC is commonly used to detect non-volatile substances in fermented mustard plant, such as organic acids and free amino acids. In their study (Liu et al., 2025), Liu et al. identified six organic acids in mustard plant using this method, including oxalic, DL-malic, L-lactic, acetic, citric, and succinic acids. Notably, L-lactic and acetic acids were found to be the primary organic acids produced during fermentation (Guan et al., 2022). Similarly, Xiao et al. (2018) reached the same conclusion when analyzing Sichuan pickled vegetables (SCP). Beyond HPLC, liquid chromatography-mass cytometry (LC–MS/MS) can also be employed for detecting organic acid content. As an integrated technique combining the strengths of liquid chromatography and mass spectrometry, LC–MS/MS provides a robust, accurate, and cost-effective method for quantitative single-generation metabolite analysis. It is considered a particularly versatile and valuable approach for analyzing lipids, carbohydrates, biogenic amines, vitamins, and organic acids (Becker et al., 2012). Liu et al. (2024) detected 63 types of organic acids in fermented vegetables through LC–MS/MS, with lactic acid and succinic acid being the predominant organic acids in fermented mustard plant-a finding consistent with the research by Liu et al. (2025).

Free amino acids (FAAs) were also detected using HPLC in the experiments carried out by Zhang et al. (2021) and Wang R. et al. (2024) revealing 17 FAAs present in fermented mustard plant. These FAAs primarily contribute to four taste profiles: umami, sweet, bitter, and neutral (Ye et al., 2022). Most studies indicate that glutamic (Glu) and aspartic (Asp) are key contributors to the umami characteristics of fermented mustard plant products, while threonine (Thr), serine (Ser), glycine (Gly), alanine (Ala), and methionine (Met) provide sweetness. Tyrosine (Tyr), leucine (Leu), isoleucine (Ile), valine (Val), phenylalanine (Phe), lysine (Lys), histidine (His), and arginine (Arg) contribute to bitterness, with cysteine (Cys) being less abundant in neutral-tasting varieties (Liu et al., 2025; Wang R. et al., 2024). While most experiments employ HPLC methods, Li J. et al. (2023) utilized UHPLC to analyze amino acid content in fermented mustard plants. Although both UHPLC and HPLC share similar working principles, UHPLC demonstrates superior sensitivity and separation efficiency, making it more suitable for high-throughput and sensitive sample analysis. However, due to the higher costs of UHPLC equipment and consumables, coupled with stringent operational requirements, HPLC remains the preferred method in most standard laboratory settings.

Through these advanced technical analyses, the researchers were able to comprehensively evaluate the flavor characteristics and sensory qualities of the detected flavor ingredients, thereby screening out LAB strains that were superior to fermented mustard plant.

### 2.3 Identification

In microbial studies of fermented mustard plant, identifying dominant bacterial strains is a crucial step. Traditional methods include morphological identification and biochemical characterization. However, due to the simplicity and low accuracy of morphological methods, as well as the limited resolution of biochemical techniques, molecular biology approaches have emerged to address these limitations in traditional methods. Currently, high-throughput sequencing technologies such as Illumina Miseq, 16S rDNA/16S rRNA sequence analysis, and metatranscriptomics are widely applied in the microbial community analysis of fermented foods, providing efficient and accurate methods for the identification of dominant strains (Table 2).

**Table 2:** Bacterial identification methods.

Strain identification	Dominant strain	Characteristics	References
High-throughput screening	*Lactiplantibacillus plantarum* CS8	1. High acid-producing capability 2. Low nitrite content enhances safety 3. Strong salt tolerance	Liu et al. (2025), Chen et al. (2006)
	*Saccharomyces cerevisiae* CX1	Enhance aroma	
16S rRNA sequencing	*Weissella* LAB1	1. Robust acid-producing ability 2. High salt and acid resistance 3. Good antibacterial capacity 4. High antioxidant capacity	Kong et al. (2025), Hou et al. (2023)
	*Leuconostoc mesenteroides* LAB2	High nitrite degradation capability	
Third-generation high-throughput sequencing	*Lactobacillus fermentum*	1. Regulate gut microbiota 2. Anti-inflammatory	Li L. L. et al. (2024)
	*Lactobacillus delbrueckii*	Good antibacterial activity	
	*Lactobacillus coryneformis*	1. Degrade ethyl carbamate (EC) 2. Reduce nitrite content 3. Improving food safety	
16SrRNA sequencing	*Lactiplantibacillus plantarum* SYS-2、SYS-4	1. High nitrite degradation efficiency 2. Fast acid production rate 3. High acid resistance and salt tolerance 4. Good antibacterial effect	Xiong Y. B. et al. (2024)
Illumina Miseq high-throughput sequencing, 16S rRNA sequencing	*Lactobacillus plantarum* GZ-2, *Lactobacillus brevis* SC-2	1. Reduce the content of BAs and nitrites 2. Improving the safety of fermented foods	Yu et al. (2021)
Molecular biology identification	*Lactiplantibacillus plantarum* LJ036	1. The strongest antibacterial capability 2. High nitrite degradation rate	Lai et al. (2026)
	*Lactiplantibacillus plantarum* LJ065	1. Robust acid-producing ability 2. The highest nitrite degradation rate 3. High antioxidant capacity	
	*Lactiplantibacillus plantarum* QT038	1. High acid resistance and salt tolerance 2. Possesses excellent growth and fermentation characteristics	

In studies of fermented vegetables, next-generation sequencing (NGS) technology is commonly employed to analyze microbial community structures (Sarengaowa et al., 2024). Studies have also focused on Illumina Miseq high-throughput sequencing, an NGS-based technology that utilizes SBS (sequencing-on-assemble) to detect sequences through PCR amplification and fluorescence signal detection (Nelson et al., 2014). Jiang et al. (2023) conducted microbial community analysis on naturally fermented and inoculated mustard plant, revealing that *Firmicutes* and *Deformata* dominated at the phylum level. At the genus level, *Bacillus*-like LAB remained predominant regardless of fermentation mode. Beyond bacterial analysis, this method also applies to fungal community composition. Fungus-level analysis identified *Ascomycota* and *Basidiomycota* as dominant fungal groups. Different fermentation methods may result in varying dominant bacterial strains during the fermentation of mustard plant, possibly due to the addition of LAB as a fermentation agent in the inoculated mustard plant (Jiang et al., 2023). Xiao et al. (2020). noted that *Lactobacillus* predominates in nearly all samples, while *Weissella* is mainly found in fermented cabbage, consistent with Kong et al.’s findings. They proposed that *Weissella* and *Lactobacillus* are dominant genera in kimchi microbial communities, each performing distinct roles during different fermentation stages to synergistically drive the process. These discoveries align with characteristic microbial profiles observed in kimchi production (Zhou Q. et al., 2023; Zhao et al., 2021). Wang et al. (2025) LAB were identified as the dominant species in the fermentation process, including *Lactiplantibacillus*, *Latilactobacillus*, *Companilactobacillus*, *Leuconostoc*, *Levilactobacillus*, and *Weissella*. Notably, *Lactiplantibacillus plantarum* and *Lactobacillus sakei* demonstrated higher abundance during fermentation, findings consistent with those reported by Correa-Galeote et al. (2022) and Salas-Millan et al. (2022). Fermentation is a dynamic process, resulting in varying dominant LAB at different stages. Xiao et al. (2018) demonstrated that at the community level, Sichuan pickled vegetable fermentation begins with environmental microorganisms (*Micrococcaceae*), progresses to an isolactic fermentation phase dominated by *Leuconostocaceae*, and concludes with a homolactic fermentation phase led by *Lactobacillaceae*. This sequence likely occurs because environmental microorganisms exhibit minimal growth during the initial phase, which ceases as environmental conditions deteriorate and LAB populations increase, thereby leading to distinct dominant LAB at different fermentation stages.

The 16S rDNA sequence analysis is a sequencing method that amplifies 16S rDNA and conducts sequence comparisons, widely used in the classification and identification of LAB. Xiao et al. (2018) demonstrated that at the phylum level, Firmicutes and Proteobacteria were the dominant phyla throughout the fermentation process of Sichuan mustard plant, consistent with Jiang et al.’s experimental findings. At the genus level, *Lactiplantibacillus plantarum* and *Lactobacillus pentosaceus* were identified as predominant strains. Lin et al. detected two new bacterial strains through phenotypic and genotypic testing, both confirmed to belong to the genus *Lactobacillus*: *Lactobacillus suantsaicola* sp. *nov.* (R7 = BCRC 81127 = NBRC 113530) and *Lactobacillus suantsaiihabitans* sp. *nov.* (R19 = BCRC 81129 = NBRC 113532) (Lin et al., 2020). The 16S rRNA sequencing technology, a molecular biology-based technique for bacterial identification and classification, has been adopted by many researchers for LAB strain characterization using primers (Martinez et al., 2013). In an experimental study by Sakai et al. (2013), partial 16S rRNA sequencing was employed to conduct taxonomic analysis on representative strains from all groups, identifying eight LAB: *Enterococcus faecium*, *Leuconostoc mesenteroides*, *Lactobacillus sakei*, *Lactobacillus curvatus*, *Lactiplantibacillus plantarum*, *Lactobacillus brevis*, *Lactobacillus parabrevis*, and *Pediococcus parvulus*. Notably, *Leuconostoc mesenteroides* and *Lactobacillus delbrueckii subsp. bulgaricus* shared identical sequences, while *Lactobacillus sakei* and *Lactobacillus curvatus*, despite their similar 16S rRNA gene sequences, exhibited distinct ratios of L-lactate to D-lactate content. In a separate study by Ren et al. (2019), 231 bacterial strains isolated from fermented foods were analyzed using 16S rRNA sequencing, revealing seven LABs with antimicrobial activity, all belonging to the *Lactobacillus* genus. Lee et al. (2020) demonstrated through 16S rRNA sequence analysis that microbial community structures in vegetables differ significantly between natural fermentation, inoculation fermentation, and commercial fermentation methods. These results indicate that both natural fermentation (which relies on the host plant’s native microbial community) and inoculation fermentation (controlled by exogenous bacterial strains) exert substantial effects on microbial community succession during kimchi production. Consequently, the predominant bacterial species identified in different studies often vary depending on the fermentation approach employed. While 16S rDNA and 16S rRNA are two distinct molecular marker analysis techniques, they are closely related in practical applications. Although direct sequencing of 16S rRNA is commonly used for sequence analysis, the high homology of the 16S rRNA gene among different bacterial species often complicates accurate classification. Therefore, in practice, indirect analysis through 16S rDNA sequencing is predominantly employed (Yang et al., 2014).

In modern fermented vegetable research, while DNA or RNA-based metagenomics can reveal microbial community structures and their genetic potential, it struggles to accurately determine which microorganisms are truly active or directly reflect their actual physiological functions during fermentation. To gain a more comprehensive understanding of the specific roles of microorganisms in food fermentation and human health, metatranscriptomics is gradually emerging as a crucial technical approach. This technology effectively identifies active members within microbial communities and their functional expression, providing new research pathways for deciphering microbial dynamics, metabolic characteristics, and the mechanisms of flavor compound formation during fermentation (Ojala et al., 2023). Nam et al. (2009) demonstrated that during the traditional Korean kimchi fermentation process, 39 LAB were tested. Metagenomic analysis revealed only 23 species were associated with kimchi fermentation, while metatranscriptomic studies identified 37 strains involved in the process. Notably, metagenomic analysis showed stable relative composition of dominant LAB, whereas metatranscriptomic analysis revealed significant variations in their relative abundance during fermentation. This indicates that metatranscriptomics can detect microorganisms with low abundance in microbial communities that participate in kimchi fermentation with high activity. In addition, studies have demonstrated that metatranscriptomic sequencing technology has been employed to elucidate the metabolic processes of microbial precursors and the pathways involved in flavor formation during food fermentation, such as the fermentation of alcoholic beverages, sauces, vegetables, and fruits. A diverse population of microbes has been identified as contributing to flavor formation (Butowski et al., 2025). Qin et al. (2024) employed metatranscriptomics and metabolomics technologies to analyze the key flavor characteristics and core functional microorganisms involved in the flavor formation of sour bamboo shoots. The study demonstrated that five genera (including *Lactococcus*, *Enterococcus*, *Leuconostoc*, *Lactiplantibacillus* and *Weissella*) actively produce enzymes related to flavor synthesis, serving as the core functional microorganisms responsible for the flavor formation of sour bamboo shoots. Xiong S. J. et al. (2024) demonstrated that the distinctive flavor profile of Laotan Suancai is governed by its unique fermentation microbial community. Through metatranscriptomic analysis, the study revealed variations in active microbial communities across fermentation stages, identifying organic acids as the primary driver of microbial succession. Furthermore, by reconstructing the metabolic network responsible for characteristic flavor compounds, the researchers identified *Companilactobacillus alimentarius*, *Weissella cibaria*, *Lactiplantibacillus plantarum*, and *Loigolactobacillus coryniformis* as core functional microbes involved in flavor formation. The study by Xiao et al. (2022) utilized metatranscriptomics to identify 27 metabolic pathways potentially associated with the production of flavor, aroma, and taste compounds of significant importance to human consumers. These pathways include those involved in fatty acid biosynthesis, the metabolism and biosynthesis of VOCs and biosynthesis, amino acid catabolism, sulfur transfer and metabolism-related pathways, and carbon metabolism pathways. Additionally, the study demonstrated that amino acids serve as crucial metabolites and flavor precursors during the fermentation of Sichuan pickles. Metatranscriptomics not only identifies the species and activity of dominant LAB during pickle fermentation but also elucidates the sources of characteristic flavors in pickles by analyzing their metabolic pathways, providing molecular-level evidence for enhancing pickle flavor and optimizing fermentation processes.

Illumina Miseq high-throughput sequencing is suitable for various types of sequencing, offering advantages such as high throughput, flexibility, and low cost (Fusco et al., 2023), making it ideal for detecting low-abundance microorganisms. 16S rDNA sequencing is applicable to studies of unculturable microorganisms, but due to its high cost and limited detection range, researchers typically combine two technologies in experiments. Zhang et al. (2021) used 16S rDNA sequencing to identify bacterial-level microorganisms, identifying *Lactobacillus sakei*, *Lactobacillus curvatus*, *C. alimentarius*, *Lactobacillus brevis*, *Lactobacillus plantarum*, and *Weissella* helenica as the dominant LAB species. Meanwhile, high-throughput sequencing was employed to identify fungal-level microorganisms, revealing *Debaryomyces debaryensis* from the genus *Halophilic Yeast* as the predominant strain during pickling fermentation. In practical applications, Illumina Miseq high-throughput sequencing is commonly used in 16S rDNA sequencing to achieve high-throughput and low-cost microbial community analysis.

Morphological identification and physico-biochemical characterization offer straightforward procedures with relatively low costs. However, their limited accuracy and resolution typically restrict these methods to preliminary screening and common bacterial genus identification. Existing research applying either morphological or physico-biochemical approaches alone remains scarce. While molecular biology techniques provide high-precision identification with superior resolution, they demand higher operational costs and technical expertise for complex or difficult-to-culture strains. To enhance accuracy, reliability, and cost-effectiveness, researchers often combine traditional methods with emerging technologies. Chen et al. (2018) exemplified this approach by conducting a preliminary classification of *Lactobacillus* isolates through morphological analysis and biochemical characterization, followed by validation via 16S rDNA gene sequencing.

Through the identification of sequencing methods, the selected dominant strains include *Lactiplantibacillus plantarum*, *Bacillus*, *Weissella*, *Leuconostoc mesenteroides*, and *Lactobacillus brevis*, as well as *Debaryomyces* and *Saccharomyces cerevisiae*. However, *Lactobacillus* remains the primary dominant strain in vegetable fermentation. This may be attributed to its significant advantages throughout the process: it not only reduces pH levels to create an optimal fermentation environment for mustard plants while developing their distinctive flavor but also inhibits the growth of spoilage bacteria, thereby ensuring both the quality and safety of fermented vegetables. However, the dominant strains of *Brassica juncea (L.)* in different samples vary, which is mainly related to the environmental conditions of the raw material origin, processing methods and fermentation parameters, microbial community detection techniques, and differences in different fermentation stages. These variations directly alter the microbial growth microenvironment, thereby selecting out more adaptable specific microbial communities.

## 3 Progress in the dominant strains

### 3.1 Lactic acid bacteria

LAB is the dominant microbial community in fermented mustard plant, including *Lactiplantibacillus plantarum*, *Lactobacillus brevis*, and *Weissella*. Such microorganisms are recognized as probiotics and play an important role in promoting human health (Guan et al., 2022). LAB inoculation in mustard plant not only improves the safety of the product (Lee K. W. et al., 2016), but also contributes significantly to the flavor formation of the fermentation system (Liao et al., 2022).

#### 3.1.1 Lactiplantibacillus plantarum

*Lactiplantibacillus plantarum* is a Gram-positive LAB widely found in fermented food. Its metabolic activity can affect the acidification rate, flavor formation, and safety guarantee of products, so it is often used as the dominant strain in the fermentation system. Compared with natural fermentation, *Lactiplantibacillus plantarum* selected by Zeng et al. (2025) showed faster acid accumulation and shorter fermentation maturity in mustard plants. It helps to form an anaerobic environment for the microbial community, while inhibiting the growth of undesirable microorganisms (Chien et al., 2023). In addition, *Lactiplantibacillus plantarum* effectively inhibited the “nitrite peak” through the degradation of autoacids and enzymes, reducing the content of nitrite, and thus, improving the safety of fermented products (Lee K. W. et al., 2016). Yu et al. (2021) selected *Lactiplantibacillus plantarum* from Sichuan samples, which were also confirmed to have this characteristic. Regarding flavor, *Lactiplantibacillus plantarum* can metabolize and produce more volatile flavor substances (such as esters, alcohols, and acids), which is conducive to enhancing the overall sour and umami taste of mustard plants and improving the acceptability of the product (Zhao et al., 2022). Wang et al. (2025) showed that *Lactiplantibacillus plantarum* not only secretes rich protease and peptidase for efficient catalytic protein degradation to dipeptides and free amino acids but can also decompose carbohydrates to produce a large number of organic acids; thus, the formation of an acidic environment further promotes the proteolysis for peptides and FAAs flavor precursor substances. It is through this dual metabolic mechanism that *Lactiplantibacillus plantarum* plays a central role in the formation of the unique flavors of fermented mustard plants.

#### 3.1.2 Weissella

*Weissella* is a genus of Gram-positive bacteria belonging to the phylum Firmicutes at the bacterial class level (Fusco et al., 2023). Due to its strong acid-producing capacity, acid and salt tolerance, excellent antibacterial properties, and antioxidant capabilities, it has become one of the dominant strains in the production of fermented mustard plant. Kong et al. isolated the dominant strain *Weissella* LAB1 from samples of mustard plant in Ningbo, Zhejiang Province. Research revealed that this strain exhibits outstanding acid production and acid resistance properties. Rapidly lowering the pH value can shorten fermentation cycles and effectively inhibit pathogenic and spoilage bacteria growth in fermented mustard plant. Compared with other samples, mustard plant inoculated with *Weissella* demonstrated better salt tolerance in saline solutions (Kong et al., 2025). The superior antibacterial activity of *Weissella* is reflected in its synthetic antimicrobial peptides, which show efficacy against both Gram-positive and Gram-negative bacteria. In Kong’s study, *Weissella* exhibited the strongest inhibitory effects on *Staphylococcus aureus* and *Escherichia coli* in fermented mustard plant samples (Kong et al., 2025). Dey et al. (2019) further demonstrated *Weissella’s* antibacterial activity against foodborne pathogens, including *E. coli*, *Listeria monocytogenes*, *Salmonella typhi*, and *Bacillus cereus.* These findings indicate *Weissella’*s effective bacteriostatic properties. Regarding antioxidant capacity, Yu et al. discovered that *Weissella* JW15 exhibits antioxidant effects through scavengers such as DPPH, ABTS, and hydroxyl radicals, demonstrating significant antagonistic and antioxidant activities (Yu et al., 2019). Similarly, in a study by Kong et al. (2025) *Weissella* demonstrated strong antioxidant capacity. The inoculation of LAB1 strains enhanced the antioxidant activity of MRS medium, which may be related to the production of antioxidant peptides. Regarding flavor development, *Weissella* produces various metabolites during sauerkraut fermentation, including organic acids, ethanol, and carbon dioxide, contributing to the improved flavor and texture of fermented vegetables (Dey et al., 2019).

#### 3.1.3 Lactobacillus brevis

*Lactobacillus brevis* is a Gram-positive bacterium characterized by its thick cell wall containing abundant peptidoglycan. Due to its strong acid-producing capacity and antibacterial properties, it has become one of the dominant strains in fermented vegetable production. According to Thomas (2018), *Lactobacillus brevis* produces organic acids such as lactic, acetic, and succinic acids, which not only aid in food acidification but also inhibit spoilage bacteria growth. The bacterium generates antimicrobial peptides that suppress certain Gram-positive and Gram-negative bacteria (Perez et al., 2014). Uymaz et al. (2011) demonstrated that *Lactobacillus brevis* isolated from traditional Turkish Tulum cheese inhibits the growth of foodborne pathogens, including *Listeria monocytogenes*, *Clostridium botulinum*, *Staphylococcus aureus*, and *Bacillus cereus*. This inhibition ensures the safety of the fermented product. Furthermore, Lim and Im (2012) research revealed that MLK27, an *Lactobacillus brevis* strain isolated from mustard plant, successfully colonizes the human intestinal tract. It exhibits the strongest adhesion inhibition against *Listeria monocytogenes* and produces organic acids, hydrogen peroxide, and bacteriocins, effectively preventing pathogenic *Listeria monocytogenes* infections. Consequently, it is recognized as a probiotic strain. As a crucial LAB with excellent fermentation capabilities, *Lactobacillus brevis* remains a key focus in food fermentation research (Thomas, 2018).

### 3.2 Yeasts

In addition to the role of LAB, the process of fermenting mustard plants is accompanied by the evolution of yeasts. The study of Kim J. Y. et al. (2022), and Kumar et al. (2022) has confirmed that *Saccharomyces cerevisiae* and *Pichia manshurica* make an important contribution to the fermentation system. Regarding flavor formation, the alcohols produced by yeast metabolism react with organic acids to form aromatic esters, which increases the content of ester flavor substances and increases the complexity of fragrance (Li et al., 2022). While promoting the esterification reaction, it accelerates the production of acid and endows fermented mustard plants with a more prominent sour taste (Xiao et al., 2023). Comprehensive studies have reported that yeasts are usually co-fermented with LAB to improve the overall fermentation performance (Boudaoud et al., 2021). Kamda et al. (2015) showed that yeasts can reduce the pH value and increase the total acid content, effectively inhibiting the growth of harmful microorganisms and greatly improving food safety. Through their unique protease systems, yeasts can hydrolyze proteins and generate small-molecule peptides and amino acids. These metabolites provide favorable conditions for the growth and reproduction of LAB, which can significantly increase the number of viable bacteria of LAB in the fermentation system (Rai et al., 2019). Relatively few studies have used yeasts alone as a dominant fermentation strain.

### 3.3 Summary

*Lactiplantibacillus plantarum*, *Weissella*, *Lactobacillus brevis*, and yeasts each demonstrate distinct advantages during the fermentation of mustard plant, playing complementary roles at different stages to form the dominant microbial community. *Lactiplantibacillus plantarum* exhibits strong acid-producing capacity, significantly increasing volatile compounds like lactic acid that enhance the flavor profile of fermented mustard plant. Additionally, it demonstrates robust nitrite degradation capabilities, effectively reducing nitrite levels in fermentation products. *Weissella*, with high abundance, collaborates synergistically with other strains during fermentation, showcasing strong acid production, acid and salt tolerance, as well as antimicrobial and antioxidant properties that make it a key contributor. *Lactobacillus brevis* inhibits pathogenic microorganisms associated with foodborne diseases, thereby enhancing the safety of fermented mustard plant. Yeasts primarily contribute to aroma formation through symbiotic fermentation with lactobacilli, endowing the product with its distinctive flavor and texture. These strains not only excel in fermentation performance but also demonstrate significant advantages in flavor, nutrition, safety, and functional characteristics. However, most experimental studies have identified lactobacilli as the predominant strains from regional samples of fermented mustard plant, leading us to conclude that lactobacilli represent the optimal microbial candidates for this fermentation process.

## 4 Analysis of the dominant strains’ fermentation mechanism

### 4.1 Acid production

**Figure 2:**
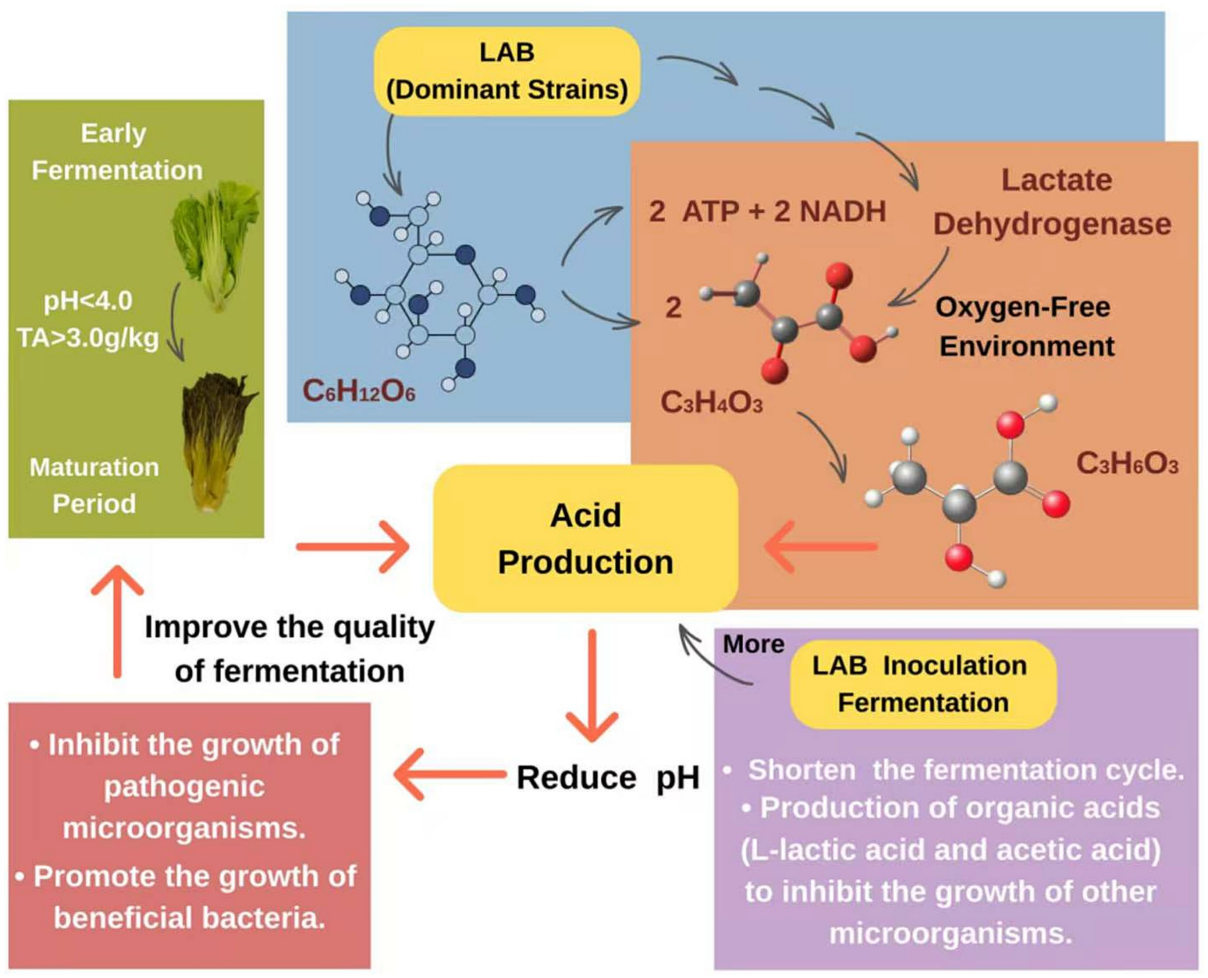
The acid production mechanism of LAB in fermented mustard plant. LAB refers to lactic acid bacteria; C_6_H_12_O_6_ refers to glucose; C_3_H_4_O_3_ refers to pyruvate; C_3_H_6_O_3_ refers to lactic acid.

TA and pH are key indicators affecting the flavor of fermented mustard plant and evaluating the maturity of fermented mustard plant, and are usually considered mature when their pH is below 4.0 and their TA content exceeds 3.0 g/kg (Yu et al., 2023). It was found that the acid production ability of fermented mustard plant mainly depends on the metabolic activity of LAB in the fermentation process (Jiang et al., 2023). LAB produces large amounts of lactate through the rapid metabolism of reducing sugars, which significantly reduces environmental pH, thus inhibiting the growth of spoilage and pathogenic microorganisms, while promoting the reproduction of other probiotics (Dalié et al., 2010; Adesulu-Dahunsi et al., 2020). Okoye et al. (2022) also confirmed that the reduction in pH contributes to the unique flavor of mustard plant and limits the contamination of non-fermented bacteria. You et al. (2025) found that the inoculation of *Lacticaseibacillus paracasei* in mustard plant contributed to the production of organic acids and bacteriocins, thus reducing pH and antagonizing other microorganisms. Similarly, Zeng et al. (2025) and Liu et al. (2025) showed enhanced acid production after inoculation with specific *Lactiplantibacillus plantarum*, which significantly shortened the fermentation cycle and improved the acid production efficiency. It follows that as the fermentation proceeds, the LAB gradually become the dominant strains in the low pH environment, further accelerating the accumulation of lactic acid (Figure 2). Of these, L-lactic acid and acetic acid are the main organic acids produced during the fermentation process (Shang et al., 2022). It is worth noting that with the fermentation of mustard plant, the lactic acid content increases with the decrease in sugar concentration (Liu et al., 2021). Zheng et al. (2020) proved that LAB can use sugar as a substrate for producing lactic acid as the main final product, so as to achieve the energy for the fermentation of mustard plant. A high salt environment or lower pH may inhibit the growth and acid production capacity of LAB, but under suitable conditions (e.g., salt environment ≦ 6%), LAB grow steadily and continue to produce acids. During late fermentation, the total acid stabilized due to inhibition of the metabolic activity of LAB (Blana et al., 2014).

### 4.2 Nitrite degradation

**Figure 3:**
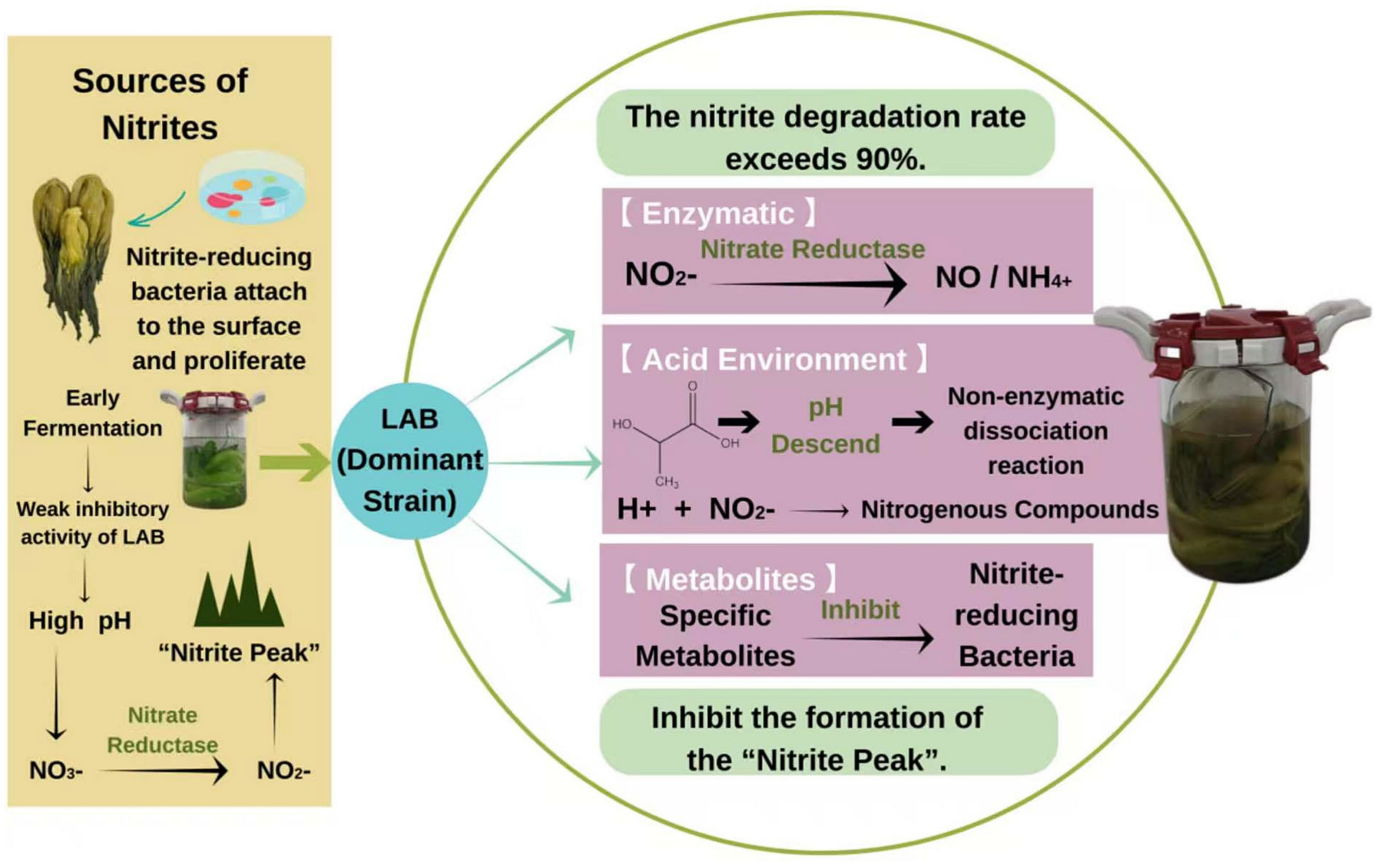
The nitrite degradation mechanism of LAB in fermented mustard plant.

Nitrite is common in all kinds of fermented vegetables, especially leafy vegetables, which is a major safety risk factor (Zhang J. et al., 2022). At the early stage of fermentation, some nitrate-reducing bacteria attached to the surface of the raw material multiply and convert the nitrate into nitrite, thus forming the “Nitrite Peak” (Ding et al., 2018). This is mainly attributed to the higher pH of the initial stage fermentation system, resulting in the weak inhibition of LAB on nitrate-reducing bacteria. To improve the food safety of fermented vegetables, many scholars aim to inoculate the dominant strains to promote the fermentation quality of vegetables. In particular, it is widely believed that LAB can degrade nitrite in three ways: the enzyme catalysis of nitrite reductase (NiR), through the acidic environment, and through specific metabolites, among which enzyme catalytic degradation is the most important pathway (Zhang X. Z. et al., 2022). Yan et al. (2008) found that LAB, as a pure starter for inoculation in cabbage, rapidly reproduced and inhibited the growth of nitrate-reducing bacteria, thus reducing nitrite accumulation. Similarly, the dominant LAB selected in fermented mustard plant exhibited more than 90% nitrite degradation rate (Saez et al., 2018). As fermentation progresses, LAB produces large amounts of lactic acid, promoting non-enzymatic disproportionality between H^+^ and NO^2−^, thus leading to the formation of nitrogen-containing compounds. This acidification further inhibited the growth of nitrite-producing bacteria, effectively blocking the formation of a “nitrite peak” in Wu (2023). In conclusion, nitrite degradation mainly depends on the multiple effects of LAB, and the inoculation of the dominant LAB is important to ensure the safety of mustard plant (Figure 3).

### 4.3 Biogenic amines degradation

Biogenic amines are a class of basic nitrogenous organic compounds with aliphatic, aromatic, or heterocyclic structures, primarily including tyramine, histamine, putrescine, cadaverine, spermine, spermidine, tryptamine, and β-phenylethylamine, which are typically generated by microbial decarboxylation of corresponding precursor amino acids (such as tryptophan, ornithine, lysine, histidine, tyrosine, etc.) (Lorenzo et al., 2007; Li L. et al., 2019). Although biogenic amines play crucial physiological roles in regulating the nervous system, cardiovascular function, and body temperature, excessive intake of foods containing high concentrations of biogenic amines may lead to various health hazards. Among these, tyramine and histamine are considered the most toxic due to their pronounced toxicological effects (Ladero et al., 2010). Consuming foods rich in such amines may lead to clinical symptoms such as headache, nausea, dyspnea, and heart failure. Furthermore, while polyamines such as putrescine, cadaverine, spermine, and spermidine are inherently less toxic, they may enhance the toxicity of tyramine and histamine by interfering with their metabolism. Additionally, these polyamines can react with nitrites to form carcinogenic N-nitrosamines, thereby further exacerbating safety risks (Lee et al., 2021).

The accumulation of biogenic amines in fermented foods results from the conversion of FAAs into corresponding amines by certain LAB through amino acid decarboxylase. These bacterial strains typically carry genes encoding amino acid decarboxylase in their genomes (Kim and Kim, 2014). From the metabolic pathway perspective, tyrosine, tryptophan, lysine, and histidine are converted into their respective amines through tyrosine decarboxylase (primarily from *Bacillus* and *Terribacillus*), tryptophan decarboxylase (primarily from *Weissella* and *Pediococcus*), L-lysine decarboxylase (primarily from *Delftia*, *Weissella*, *Lactobacillus*, and *Aquabacterium*), and L-histidine decarboxylase (primarily from *Weissella* and *Aquabacterium*) (Yu et al., 2022a,b).

Currently, the application of LAB (primarily *Lactiplantibacillus plantarum*, *Latilactobacillus sakei*, *Lacticaseibacillus paracasei*, etc.) in the biodegradation of bioamines in wine, fish paste, sausages, and cheese has been confirmed by multiple scholars (Capozzi et al., 2012; Li C. S. et al., 2024; Schirone et al., 2022). Its degradation mechanism primarily involves enzymes such as multicopper oxidase (MCO) gene and amine oxidase, which can oxidize biogenic amines into aldehydes, hydrogen peroxide, and ammonia (Pisteková et al., 2020). Lee et al. (2021). demonstrated through fermentation inoculation that *Lactobacillus brevis* PK08, PK11, and JCM 1170 exhibited high biogenic amine degradation activity in kimchi fermentation, confirming the pivotal role of their MCO gene in tyramine degradation. The study by Pavel et al. demonstrated that *Lactiplantibacillus plantarum* inoculation significantly inhibited the formation of tyramine, putrescine, and cadaverine in kimchi (Pavel et al., 2000). Notably, the glyceralde-hyde-3-phosphate dehydrogenase (GAPDH) in *Lactiplantibacillus plantarum* has also been demonstrated to possess histamine-degrading capability, with the combination of MCO and GAPDH genes playing a role in the β-phenylethylamine degradation of kimchi (Sun et al., 2020). This finding has certain implications for the safety of fermented mustard plant, which needs further study.

### 4.4 Texture formation

The texture of fermented mustard plant is a key indicator of its quality, which directly affects consumers’ taste acceptance. The textural properties of plant tissues are primarily determined by the integrity of cell walls and their microstructure. Pectin, as the core component of cell walls, plays a crucial role in maintaining tissue hardness (Yang et al., 2023). Pectinase are heterogeneous enzyme system capable of hydrolyzing pectin, widely distributed in higher plants and microorganisms (Jayani et al., 2005). During fermentation, pectinase secreted by microorganisms acts on the pectin components in the cell wall, degrading them into soluble monosaccharides or oligosaccharides (Purwanto et al., 2024). This enzymatic reaction leads to structural loosening or even localized rupture of the cell wall, reducing chewing resistance and thereby transforming the texture of mustard plant from hard to soft, crisp., and resilient, thereby improving the mouthfeel (Jayani et al., 2005).

As the dominant bacteria in the fermentation process of mustard plant, LAB is the main source of pectinase and other cell wall-degrading enzymes. Sarengaowa et al. (2024) demonstrated that the abundance of *Lactobacillus* species (particularly *Lactiplantibacillus plantarum* and *Lactobacillus selangorensis*) was positively correlated with the softening of mustard plant. The study by Zhang et al. (2012) further confirmed that the hardness of fermented mustard plant inoculated with LAB was lower than that of traditional fermented mustard plant, and this was attributed to the promotion of pectin conversion by LAB, whereas in traditional fermentating processes, pectin remains abundant in the cell walls, resulting in a firm and dense tissue texture. For leafy vegetables such as mustard plant, which are rich in leaf tissue, moderate cell wall degradation facilitates the softening of fibrous structures, imparting a more tender and palatable texture to the product. However, current research on the relationship between specific bacterial strains in fermented vegetables and the formation of texture mechanisms remains relatively limited.

### 4.5 Flavor regulation

#### 4.5.1 Amino acids

**Figure 4:**
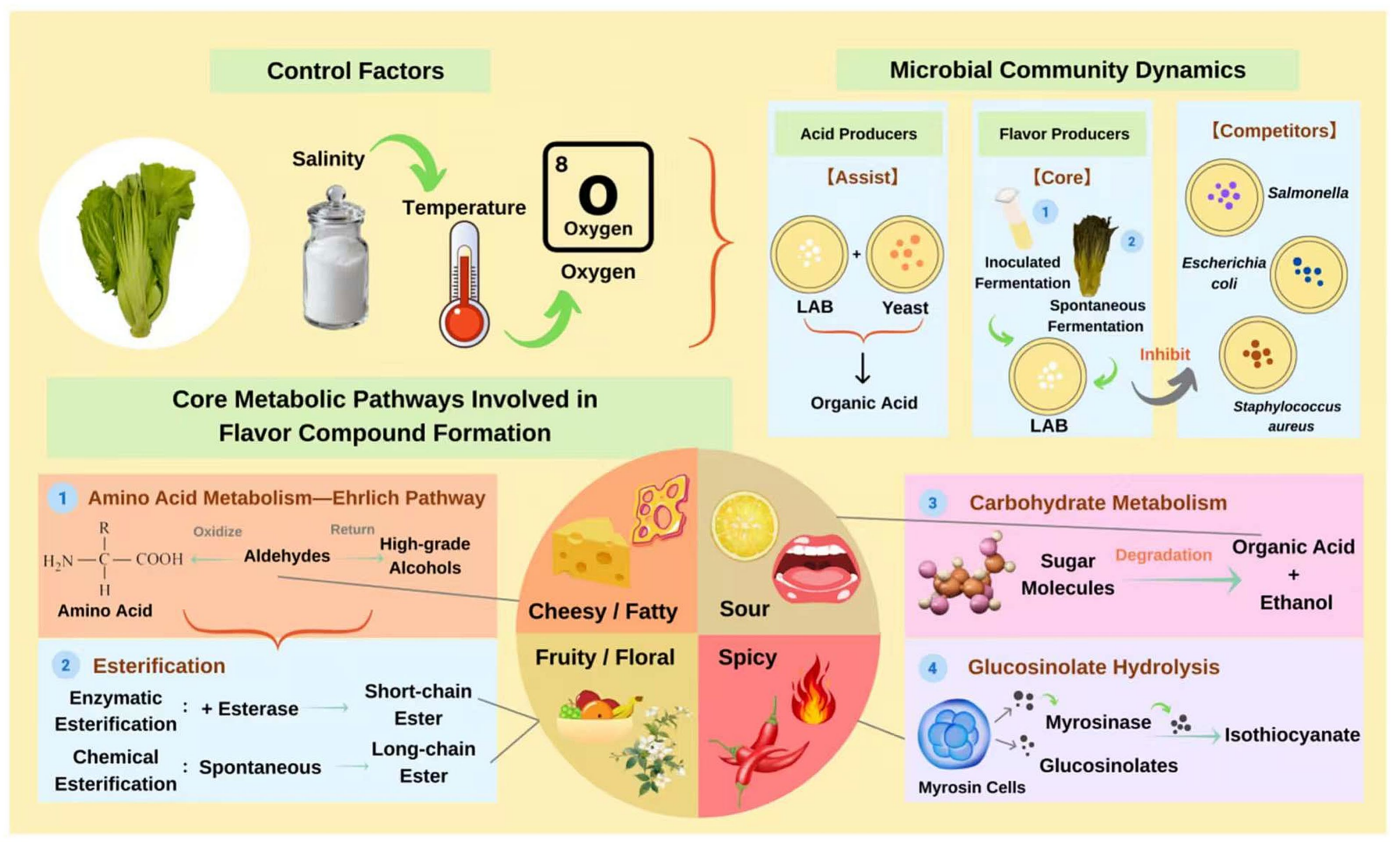
The flavor formation mechanism of fermented mustard plant.

Amino acids and their nitrogen content are important indicators of the fermented flavor of mustard plants, which is positively correlated with the flavor complexity of the final product (Kong et al., 2025). Luo et al. (2024) found that during fermentation, microbial activity degrades a large number of proteins into amino acids. This process primarily relies on the proteolytic activity of microbial enzymes in the starter strains. The experiments by Christensen et al. elucidated that among starter strains (*Lactococcus cremoris*), those carrying the *PrtP* protease were confirmed as proteolytic strains, which modify the environmental amino acid pool through extracellular proteolytic activity (Christensen et al., 2024). This not only increases the total amount of amino acids but also alters the composition of flavor precursor compounds. Abubakr and Al-Adiwish (2017) confirmed that *Lactiplantibacillus plantarum* exhibits stronger proteolytic activity in mustard plant fermentation. Zhao et al. (2023) found that amino acids dominate the whole fermentation process when comparing the changes in metabolites. The significant increase in amino acids plays an important role in the development of flavor in fermented mustard plant, facilitating the production of various aroma metabolites (such as acids, alcohols, esters, and carbonyl groups) (Zhang et al., 2021). Liu et al. (2025) compared natural fermentation with specific *Lactobacillus plantarum* inoculation fermentation and found that the content of FAAs, such as glutamine (Gln) and asparagine (Asn), increased significantly in the early stage of fermentation. This mainly stems from the degradation of the protein by the dominant strain and the elevated peptidase activity (Chen et al., 2022), and these umami and sweet amino acids directly enhance the flavor of the product (Becker et al., 2012). Further studies have demonstrated that the synergistic fermentation of flavourzyme with specific LAB can significantly increase protein degradation rates, elevate FAAs content, and accumulate sweet and umami amino acids (Xiao et al., 2024). However, as the fermentation progressed, the FAAs content gradually decreased, and some microorganisms were converted into small-molecule flavor substances (such as organic acids and volatile compounds), which further enriched the flavor hierarchy (Zhong et al., 2022). Some LAB isolated from conventional fermentation were proven to produce high Glu via protein metabolism (Sevindik et al., 2022). Glu catabolism enhances the acid tolerance of fermented mustard plant, and it is likely that this particular LAB uses glutamate dehydrogenase and glutamate decarboxylase to break down Glu, indirectly regulating flavor stability (Li et al., 2020). Liu et al. (2021) suggested that the abundance of *Weissella* and *Lactobacillus* was positively correlated with amino acids in the flavor regulation of fermented mustard plant. In conclusion, amino acids are not only direct contributors to flavor substances but also participate as precursors in the generation of other flavor compounds, thus forming the synergistic effect of fermented mustard plant’s umami, sweet and complex flavor (Figure 4) (Rai et al., 2019).

#### 4.5.2 Organic acids

Organic acids, as the core products of microbial metabolism, regulate the flavor formation of fermented foods through multiple mechanisms (Figure 4). During the whole fermentation process, the content of organic acids and their derivatives shows a dynamic trend of rising, then falling, and then rising again (Wang et al., 2025). This law of change may be derived from the staged characteristics of microbial metabolic activity. In the early stage of fermentation, microorganisms produce a large number of organic acids through nutritional metabolism. With the consumption of nutrients such as carbohydrates and proteins, microorganisms turn to the previously accumulated organic acids as a carbon source in the later stage of fermentation, resulting in a temporary decline in their content or flattening (Liu et al., 2021). Studies have found that the organic acid content may be related to the inoculated species. The inoculation of *Lactobacillus plantarum* in fermented mustard plants allows for rapid acid production, and the favorable acidic environment causes *Lactobacillus plantarum* to dominate the fermentation process, creating a more uniform flavor characteristic (Liu et al., 2025). It is noteworthy that *Lactiplantibacillus plantarum* can secrete cell wall hydrolases such as pectinase and cellulase in fermented foods (Ngamnok et al., 2023). This not only promotes the decomposition of plant tissue structure, but also releases fermentable sugars during the degradation of pectin in the cell wall. These sugars are subsequently converted into organic acids by the bacterial strains, thereby enhancing the acidity and flavor of the fermentation system. The study by Du et al. further corroborates this perspective (Du et al., 2021). Thus, compared with natural fermentation, inoculated strains (e.g., *Bacillus marcorestinctum* YC-1) can promote the accumulation of lactic acid and acetic acid by changing microbial communities to give fermented mustard plant a unique sour taste (Liang et al., 2020). Acid-producing microorganisms metabolize nutrients through glycolysis, the pentose phosphate pathway, and the TCA cycle, thus significantly increasing the content of organic acids (Zhao et al., 2021). The accumulation of organic acids not only directly confers characteristic flavors such as spicy and vinegary but also selectively inhibits the contamination of non-fermentable bacteria by reducing pH (Okoye et al., 2022). In addition, in the late stage of fermentation, microorganisms reuse organic acids as carbon sources to form a dynamic metabolic balance, while the amino acids produced by protein degradation cooperate with organic acids to further enrich the flavor hierarchy (Wang Y. Z. et al., 2022).

#### 4.5.3 Other metabolites

We have shown that the dominant strains in fermented mustard plant can promote the production of metabolites and achieve flavor regulation through the generation and transformation of various compounds (Figure 4). Specific *Lacticaseibacillus paracasei* inoculated by You et al. (2025) produced large amounts of organic acid, and higher concentrations of aldehydes, alcohols, and esters conferred a herby and fruity flavor to fermented mustard plant (Sun et al., 2023). Specific *Lactiplantibacillus plantarum* promoted the esterification reaction of acidic compounds by enhancing esterase activity, thus increasing the content of esters (Li Z. et al., 2023). The higher concentration of esters in fermented mustard plant gives it a unique aromatic odor, which may be a result of the chemical reaction between organic acids and alcohols (Zhao et al., 2024). The strong, spicy taste in fermented mustard plant is mainly derived from isothiocyanates (ITCs). Mustard plant, being rich in glucosinolates (GSLs), can be hydrolyzed into a series of thiocyanate compounds under the action of myrosinases, resulting in dominant esters such as allyl isothiocyanate, sec-butylisothiocyanate, and 3-butylisothiocyanate (Wei et al., 2021). Palop et al. (1995) found that the content of GSLs compounds decreased significantly with the fermentation process due to the hydrolysis of microorganisms and endogenous enzymes. Myrosinase, as an endogenous β-thioglucosidase, catalyzes the glycosidic bond hydrolysis of GSLs, generating thiohydroximate-O-sulfonate intermediates, which are subsequently converted into ITCs (Liang et al., 2025). However, in intact plant tissues, myrosinase and its substrate GSLs are typically spatially isolated, stored in distinct cellular structures, thereby preventing hydrolytic reactions. Salinization and associated physical inhibitory effects compromise the integrity of cellular tissues, facilitating enzyme-substrate contact and thereby effectively initiating the hydrolysis of GSLs, which promotes the formation of flavor compounds such as ITCs (Tripathi and Mishra, 2007). Similarly, Mullaney et al. (2013) and Wang M. Q. et al. (2022) found that LAB were also able to hydrolyze GSLs. The study by Liang et al. (2025) further corroborated this perspective. Through transcriptomic and proteomic analyses, their experiment identified a myrosinase gene (named LpMyr) in *Lactiplantibacillus plantarum* (*Lactiplantibacillus plantarum* ZUST49) and verified its efficacy in degrading GSLs. Plaza-Vinuesa et al. (2022) also identified a *Lactiplantibacillus plantarum* (*Lactiplantibacillus plantarum* WCFS1) with myrosinase-like enzyme activity, which could hydrolyze plant-GSL in induction medium. These results all prove that the specific LAB strain enhanced the hydrolysis because of the codification of functional active myrosinase. As fermentation proceeds, the total alcohol content of fermented mustard plant gradually increases, which may be due to carbohydrate degradation or amino acid catabolism or via fermentation from LAB and yeasts (Rajendran et al., 2023; Zhao et al., 2007). Aldehydes are divided into saturated and unsaturated aldehydes. Specifically, unsaturated aldehydes have a cheesy and fruity flavor, while saturated aldehydes have a spicy and stimulating taste (Adams et al., 2008). Aldehydes contribute greatly to the flavor of fermented mustard plant due to their low threshold (Dan et al., 2012).

### 4.6 Microbial diversity

The dynamic changes of microbial communities during fermentation and their interaction with environmental factors are important reasons for microbial diversity. Wang D. D. et al. (2022) found that in the early stage of fermentation, microorganisms in the raw materials and the environment (such as *Pseudomonas* and enterobacteria) were gradually replaced by salt-resistant microorganisms (such as *Lactiplantibacillus plantarum* and *Halomonas*) due to the double coercion of salt and acid, resulting in a decrease in bacterial diversity and abundance. This law is similar to the results of the macrogenome sequence analysis conducted by Li et al. (2020). The most characteristic group in the final stage of fermentation in mustard plant is LAB, with the highest sequence ratio (Liang et al., 2018). LAB produces a large amount of lactic acid through rapid reproduction and metabolic activities, reduces pH, produces antibacterial agents to inhibit the growth of harmful microorganisms (such as Coliform Zeng et al., 2025), and promotes the proliferation of beneficial bacteria (such as *Weissella* Yan et al., 2022), thus improving the safety and flavor of fermented products (Liu et al., 2020). Of these, *Lactobacillus* has gradually become the dominant bacteria of fermented mustard plant. This is consistent with the observations of Jung et al. (2011) and Hu et al. (2024). Among them, Liu et al. (2024), based on Illumina high-throughput sequencing, found that the main LAB in fermented vegetables are *Lactobacillus*, with the highest relative abundance. *Lactobacillus* is considered to be the core microbiota of Chinese fermented vegetables such as Jiangxi salted vegetables, Sichuan pickles, and Northeast sauerkraut, and is conducive to the formation of flavor in the fermentation process of pickles (Shang et al., 2022). *Aspergillus* is widely found in nature, and its growth may lead to the softening of vegetable tissue, which, in turn, leads to the decomposition the molding and decay of fermented vegetables (Cao et al., 2017). Inoculation fermentation further optimizes the microbial structure by introducing specific strains, inhibiting the growth of mold, and ensuring the safety of fermentation. This conclusion was verified in the experiments of Jiang et al. (2023). The diversity of fungi gradually decreases, which may be due to the failure of aerobic fungi to adapt to the anaerobic environment, resulting in their death (Jiang et al., 2023). At present, there are few studies on the abundance of fungi in fermented mustard plant. In a word, microbial diversity is jointly regulated by nutritional competition, environmental coercion, and vaccination intervention, eventually forming a functional community with LAB as the core.

## 5 Conclusion

In the fermentation of mustard plant, the isolation, screening, and identification of dominant bacterial strains are crucial processes for enhancing the quality, flavor, and safety of fermented products. Most studies have indicated that while samples of mustard plants from different regions yield various dominant strains, including LAB and yeasts, LAB play a dominant role in fermentation. The methods for isolating, screening, and identifying these strains include traditional cultivation techniques (such as streaking plates and dilution spread plates), morphological observation (Gram staining), physicochemical experiments (direct pH measurement, naphthylamine hydrochloride colorimetry, DPPH free radical scavenging, GC–MS, and HPLC), and molecular biology technologies (Illumina Miseq high-throughput sequencing, conventional 16S DNA sequencing and metatranscriptomics). LAB exhibit exceptional performance during fermentation: they not only effectively reduce pH levels, but also demonstrate strong acid production capabilities with acid and salt tolerance. Additionally, they possess excellent antioxidant capacity, antibacterial activity, and nitrite degradation ability, making them valuable for ensuring food safety and health in fermented products. Regarding flavor formation, LAB metabolize organic acids, volatile compounds, and non-volatile substances. Their low acidity inhibits harmful microbial growth, enhancing product safety. The formation of diverse flavor compounds enriches the texture and aroma of fermented mustard plant, significantly improving their overall taste profile. The screening and identification of dominant bacterial strains during mustard plant fermentation not only enhances production efficiency and product quality but also provides theoretical support for developing novel fermentation agents and optimizing fermentation processes. Future research should further integrate high-throughput screening technologies with multi-omics analysis methods to thoroughly explore the metabolic mechanisms and functional characteristics of these dominant strains, thereby advancing the industrialization and standardization of fermented mustard plant products.
